# Phylogeography and Genetic Ancestry of Tigers *(Panthera tigris)*

**DOI:** 10.1371/journal.pbio.0020442

**Published:** 2004-12-07

**Authors:** Shu-Jin Luo, Jae-Heup Kim, Warren E Johnson, Joelle van der Walt, Janice Martenson, Naoya Yuhki, Dale G Miquelle, Olga Uphyrkina, John M Goodrich, Howard B Quigley, Ronald Tilson, Gerald Brady, Paolo Martelli, Vellayan Subramaniam, Charles McDougal, Sun Hean, Shi-Qiang Huang, Wenshi Pan, Ullas K Karanth, Melvin Sunquist, James L. D Smith, Stephen J O'Brien

**Affiliations:** **1**Laboratory of Genomic Diversity, National Cancer InstituteFrederick, MarylandUnited States of America; **2**Conservation Biology Graduate Program, University of MinnesotaSt. Paul, MinnesotaUnited States of America; **3**Wildlife Conservation Society, Russian Far East ProgramBronx, New YorkUnited States of America; **4**Wildlife Conservation Society, Hornocker Wildlife InstituteBozeman, MontanaUnited States of America; **5**Minnesota Zoo, Apple ValleyMinnesotaUnited States of America; **6**Potter Park Zoo, LansingMichiganUnited States of America; **7**Singapore Zoological GardensSingapore; **8**Zoo Negara, Hulu KelangSelangorMalaysia; **9**Tiger TopsKathmanduNepal; **10**International Cooperation Office, Ministry of Agriculture Forestry and FisheriesPhnom PenhCambodia; **11**Beijing ZooBeijingChina; **12**College of Life Sciences, Peking UniversityBeijingChina; **13**Wildlife Conservation Society—India Program, BangaloreKarnatakaIndia; **14**Department of Wildlife Ecology and Conservation, University of FloridaGainesville, FloridaUnited States of America

## Abstract

Eight traditional subspecies of tiger *(Panthera tigris),* of which three recently became extinct, are commonly recognized on the basis of geographic isolation and morphological characteristics. To investigate the species' evolutionary history and to establish objective methods for subspecies recognition, voucher specimens of blood, skin, hair, and/or skin biopsies from 134 tigers with verified geographic origins or heritage across the whole distribution range were examined for three molecular markers: (1) 4.0 kb of mitochondrial DNA (mtDNA) sequence; (2) allele variation in the nuclear major histocompatibility complex class II *DRB* gene; and (3) composite nuclear microsatellite genotypes based on 30 loci. Relatively low genetic variation with mtDNA, *DRB,* and microsatellite loci was found, but significant population subdivision was nonetheless apparent among five living subspecies. In addition, a distinct partition of the Indochinese subspecies *P. t. corbetti* into northern Indochinese and Malayan Peninsula populations was discovered. Population genetic structure would suggest recognition of six taxonomic units or subspecies: (1) Amur tiger *P. t. altaica;* (2) northern Indochinese tiger *P. t. corbetti;* (3) South China tiger *P. t. amoyensis;* (4) Malayan tiger *P. t. jacksoni*, named for the tiger conservationist Peter Jackson; (5) Sumatran tiger *P. t. sumatrae;* and (6) Bengal tiger *P. t. tigris*. The proposed South China tiger lineage is tentative due to limited sampling. The age of the most recent common ancestor for tiger mtDNA was estimated to be 72,000–108,000 y, relatively younger than some other *Panthera* species. A combination of population expansions, reduced gene flow, and genetic drift following the last genetic diminution, and the recent anthropogenic range contraction, have led to the distinct genetic partitions. These results provide an explicit basis for subspecies recognition and will lead to the improved management and conservation of these recently isolated but distinct geographic populations of tigers.

## Abstract 2

Genetic analysis provides the basis for subspecies recognition among tigers, and will lead to improved conservation strategies for these endangered animals

## Introduction

The tiger *(Panthera tigris)* is the largest felid species and a widely recognized symbol of wildlife conservation. Historically tigers inhabited much of Asia, including the regions between the Caspian and Aral Seas, southeastern Russia, and the Sunda islands (Mazak 1981; Hemmer 1987; Herrington 1987). Since the early 1900s, however, habitat loss, fragmentation, and human persecution have reduced tiger populations from probably over 100,000 in 1900 to fewer than 7,000 free-ranging individuals (Nowell and Jackson 1996; Dinerstein et al. 1997; Kitchener and Dugmore 2000). Most populations consist of less than 120 animals, increasing the risk of local extirpation due to demographic and genetic factors (Smith and McDougal 1991; Dinerstein et al. 1997).

**Figure 1:**
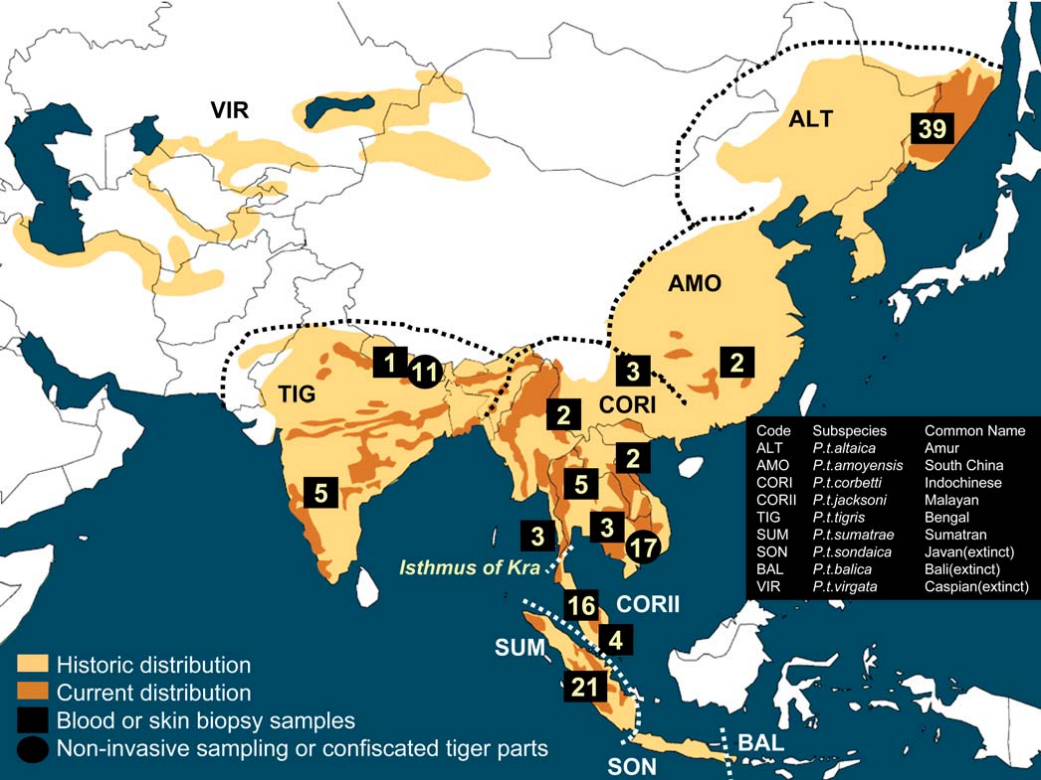
Historic and Current Geographic Distribution of Tigers Corresponding to the Eight Traditional Subspecies Designation Geographic origin of samples and sample size (circles or squares) from each location are indicated (see Table 3 for sources). Three-letter codes (TIG, ALT, etc.) are indicated subspecies abbreviations. Dotted lines are approximate boundaries between tiger subspecies studied here. The Isthmus of Kra divides the traditional Indochinese tigers into the northern Indochinese tigers *P. t. corbetti* I and the Malayan tigers *P. t. corbetti* II based on the present study. We propose the Malayan tiger subspecies, COR II, be named *P. t. jacksoni,* to honor Peter Jackson, the former Chair of the IUCN's Cat Specialist Group who has contributed significantly to worldwide tiger conservation.

There are eight generally accepted tiger subspecies in accordance with their geographic distribution (Figure 1). Bali *(P. t. balica),* Caspian *(P. t. virgata),* and Javan (*P. t. sondaica*) tiger subspecies were eradicated by the 1940s, 1970s, and 1980s respectively (Nowell and Jackson 1996). Today an estimated 3,200–4,500 Indian or Bengal tigers *(P. t. tigris)* exist in Bangladesh, Bhutan, western China, India, western Myanmar, and Nepal (Seidensticker et al. 1999). Fewer than 500 Amur or Siberian tigers *(P. t. altaica)* survive in eastern Russia, northeastern China, and Korea (Matyushkin et al. 1999; Miquelle and Pikunov 2003), while approximately 50 Amoy or South China tigers *(P. t. amoyensis)* now exist in captivity only (Tilson et al. 2004). An estimated 400–500 Sumatran tigers *(P. t. sumatrae)* occur in Sumatra (Seidensticker et al. 1999); and 1,200–1,800 Indochinese tigers *(P. t. corbetti)* live in Cambodia, China, Laos, Malaysia, east Myanmar, Thailand, and Vietnam (Seidensticker et al. 1999) (Figure 1).

**Table 3:**
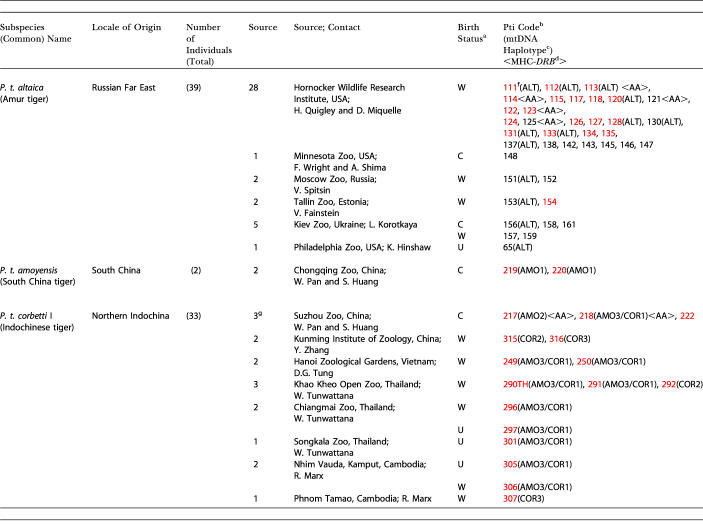
Samples of Panthera tigris Used in the Study

^a^ Birth Status of each tiger: W, wild-born, C, captive-born; U, status unknown

^b^ Identification number of tiger individuals as they are listed in the database at the Laboratory of Genomic Divesity, National Cancer Institute, Frederick, Maryland, United States

^c^ MtDNA haplotype assigned to each sample sequenced in the study

^d^ MHC ClassII *DRB* allele genotypes

^e^ Samples of pelt or hair

^f^ Red samples represent samples with microsatellite data from 30 loci

^g^ Tigers individuals classified as South China tiger originally

Subspecies of tigers are traditionally defined by body size, skull characters, pelage coloration, and striping patterns (Mazak 1981; Herrington 1987). It is generally believed that the largest tigers occur in the Russian Far East, and the smallest are found in the Sunda Islands. The shape of the occiput in the skull is characteristically narrow in the Javan and Bali tigers and much broader in Caspian tigers (Mazak 1996). However, the adequacy of these traditional subspecies designations is tentative at best, since morphological distinctions in many cases have been based on a few specimens, and because subsequent studies have failed to affirm these distinctions. Herrington (1987) and Kitchener (1999) have revealed a wide range of morphological variations within the subspecies and, to some extent, overlapping among the subspecies. A previous molecular genetic assessment of 28 tigers has indicated a low level of genetic variation, revealing little evidence for subspecies distinctiveness (Wentzel et al. 1999). Moreover, ecological analyses of tiger habitat (Kitchener and Dugmore 2000) indicate that there have been few geographic barriers (e.g., mountain ranges and deserts) to migration and gene flow that would have been sufficient for subspecies isolation. One ecology-based conservation approach emphasizes protection of about 160 continuous habitat patches or tiger conservation units regardless of subspecies designation (Dinerstein et al. 1997). Although this strategy may be desirable, optimal tiger conservation may also require additional interventions such as establishing corridors and buffer zones and/or implementing reintroduction programs (Tilson et al. 2001). To this end, an assessment of population genetic structure of living tigers interpreted in the context of traditional intraspecific taxonomy and the species' evolutionary history would benefit both in situ and ex situ conservation management design.

Molecular genetic markers have been increasingly applied to assess genetic partitions among geographically isolated populations, to define the evolutionary significant unit below the species level for conservation management purposes, and to revise the traditional species and subspecies designations (Avise and Ball 1990; Moritz 1994; Fraser and Bernatchez 2001). Subspecies recognition is particularly relevant for tigers, because the current conservation strategy for this species has been inextricably bound to knowledge of its subspecific taxonomy. In this study we adhere to the subspecies concept as defined by Avise and Ball (1990) and O'Brien and Mayr (1991), to include populations below the species level that share a distinct geographic distribution, a group of phylogenetically concordant characters, and a unique natural history relative to other subdivisions of the species.

Here we attempt to overcome several factors that have complicated previous efforts to fully describe patterns of genetic variation in tigers. Foremost among these has been the limited sample size of “voucher specimens” (defined as individuals that were verified as wild-born from a specific geographic locale or captive-born from geographically verified wild-born parents). In addition, the presence of Numt, a nuclear pseudogene insertion of cytoplasmic mitochondrial DNA (mtDNA) in tiger autosomes (Lopez et al. 1994; Johnson et al. 1996; Cracraft et al. 1998; J. H. Kim, A. Antunes, S.-J. Luo, J. Menninger, W. G. Nash, et al., personal communication) has made it difficult to utilize universal mammalian primer sets for mitochondrial genes, because they will coamplify Numt. Furthermore, paucity of genetic diversity across tigers, especially in mtDNA (Wentzel et al. 1999), has made it necessary to sequence a large portion of the mtDNA genome and to assess genetic variation in multiple rapidly evolving microsatellite loci.

To establish proper biological reference specimens, samples from 134 tigers of known geographic origin were collected. Three genetic markers were examined: (1) 4 kb of mtDNA sequence derived from primer pairs that excluded Numt amplification, (2) allele variation in the major histocompatibility complex (MHC) *DRB* gene; and (3) allele size variation of 30 hypervariable short tandem repeat loci or microsatellites. Observed patterns of population genetic variation replicated with different gene families form the basis of interpretation of the tiger's evolutionary history and recommendations for its management.

## Results

### Phylogenetic Analysis of mtDNA and Microsatellites

**Figure 2:**
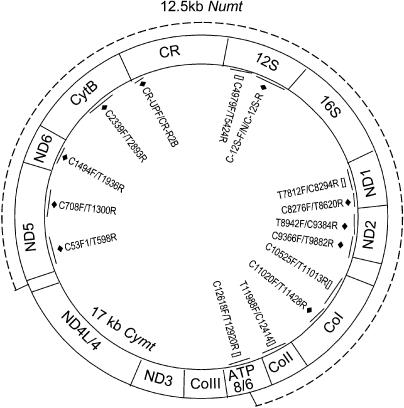
Schematic of P. tigris mtDNA The position of PCR primers used for amplification of Cymt specific sequences and alignment of the homologous Numt sequence (outer, dashed line) in tiger mitochondria. Fifteen Cymt-specific primer sets spanning 6,026 bp of mtDNA were designed and screened for polymorphism in tigers (inner, solid line). Five indicated segments showed no variation among fifteen tigers that represented five traditional subspecies and therefore were excluded from further analysis. The ten variable segments (4,078 bp) were amplified in 100 tiger individuals. Primer sequences are listed in Table 1. Diamonds indicate polymorphic mtDNA segments; brackets indicate monomorphic mtDNA segments among tigers that were excluded from phylogenetic analysis.

Mitochondrial gene fragments were amplified and sequenced from DNA extracted from 72 blood or tissue specimens using 10 cytoplasmic mitochondria (Cymt)-specific primer pairs (Figure 2 and Table 1). The fragments were concatenated in a 4,078-bp contiguous sequence. Additional mtDNA sequences were generated from 28 historical samples (pelt or hair) by amplifying shorter fragments (less than 400 bp) targeting selected variable sites to determine their similarity to the previously characterized haplotypes. Combined mtDNA sequences were obtained from 100 tigers from Russian Far East (*n* = 13), south China (*n* = 4), northern Indochina (*n* = 30), Malayan Peninsula (*n* = 22), Sumatra (*n* = 16), and the Indian subcontinent (*n* = 15). The mtDNA sequences specified 54 variable sites defining 25 haplotypes (Table 2). Thirty of the polymorphisms were observed in more than one individual and were thus phylogenetically informative (Table 2), and 29 of the 30 changes were transitions.

**Table 1:**
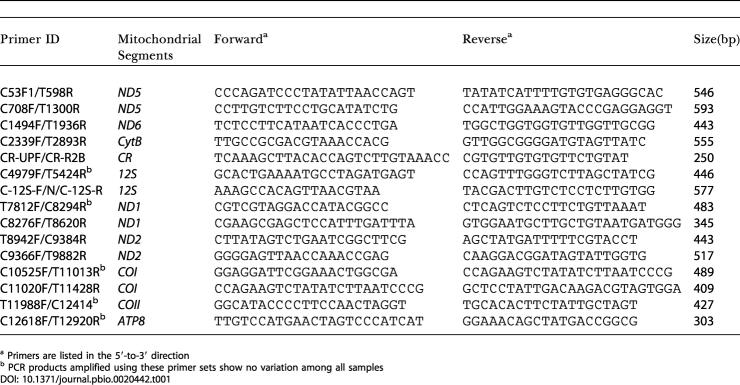
PCR Primers Specific for Cytoplasmic Mitochondrial DNA Sequences

^a^ Primers are listed in the 5′-to-3′ direction

^b^ PCR products amplified using these primer sets show no variation among all samples

**Table 2:**
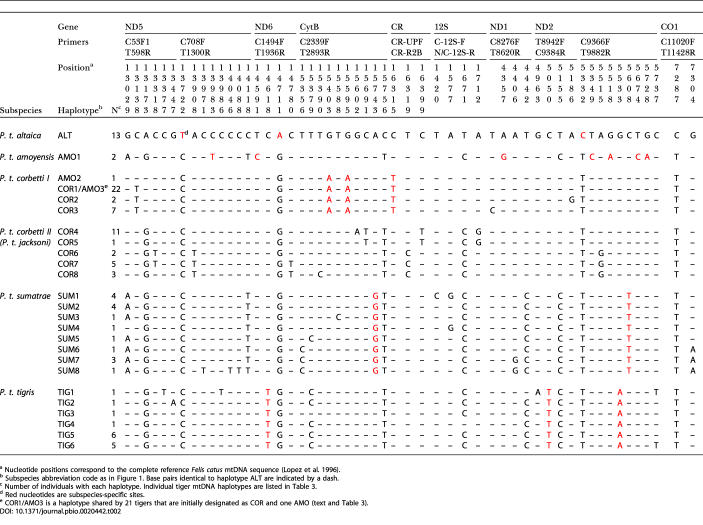
Haplotypes and Variable Sites in Combined Analysis of 4,078 bp of Tiger *(P.tigris)* mtDNA Sequences

^a^ Nucleotide positions correspond to the complete reference Felis catus mtDNA sequence (Lopez et al. 1996)

^b^ Subspecies abbreviation code as in Figure 1. Base pairs identical to haplotype ALT are indicated by a dash

^c^ Number of individuals with each haplotype. Individual tiger mtDNA haplotypes are listed in Table 3

^d^ Red nucleotides are subspecies-specific sites

^e^ COR1/AMO3 is a haplotype shared by 21 tigers that are initially designated as COR and one AMO (text and Table 3)

**Table 3:**
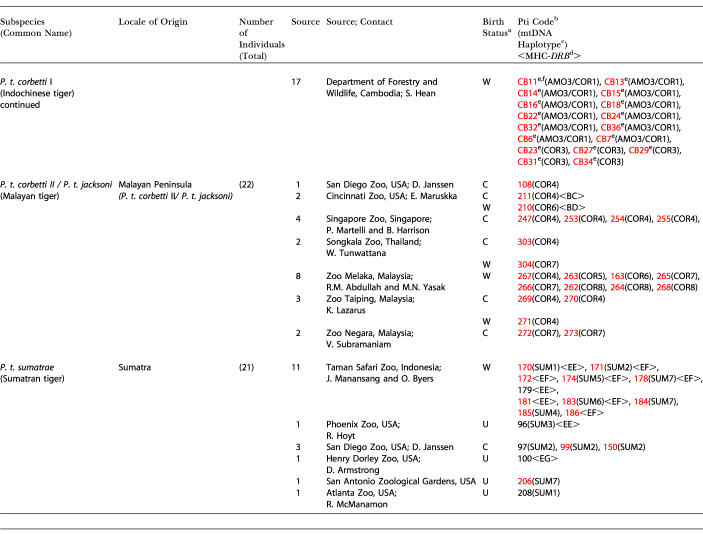
Continued

**Table 3:**
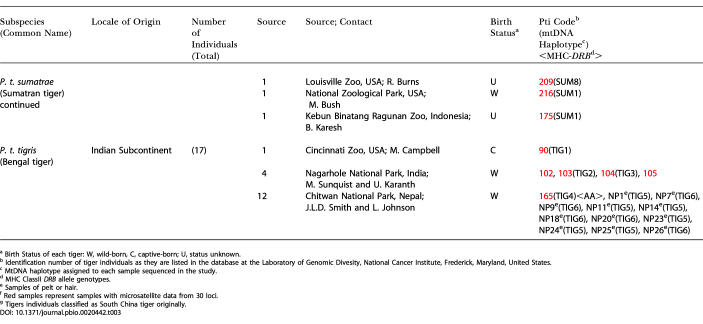
Continued

**Figure 3:**
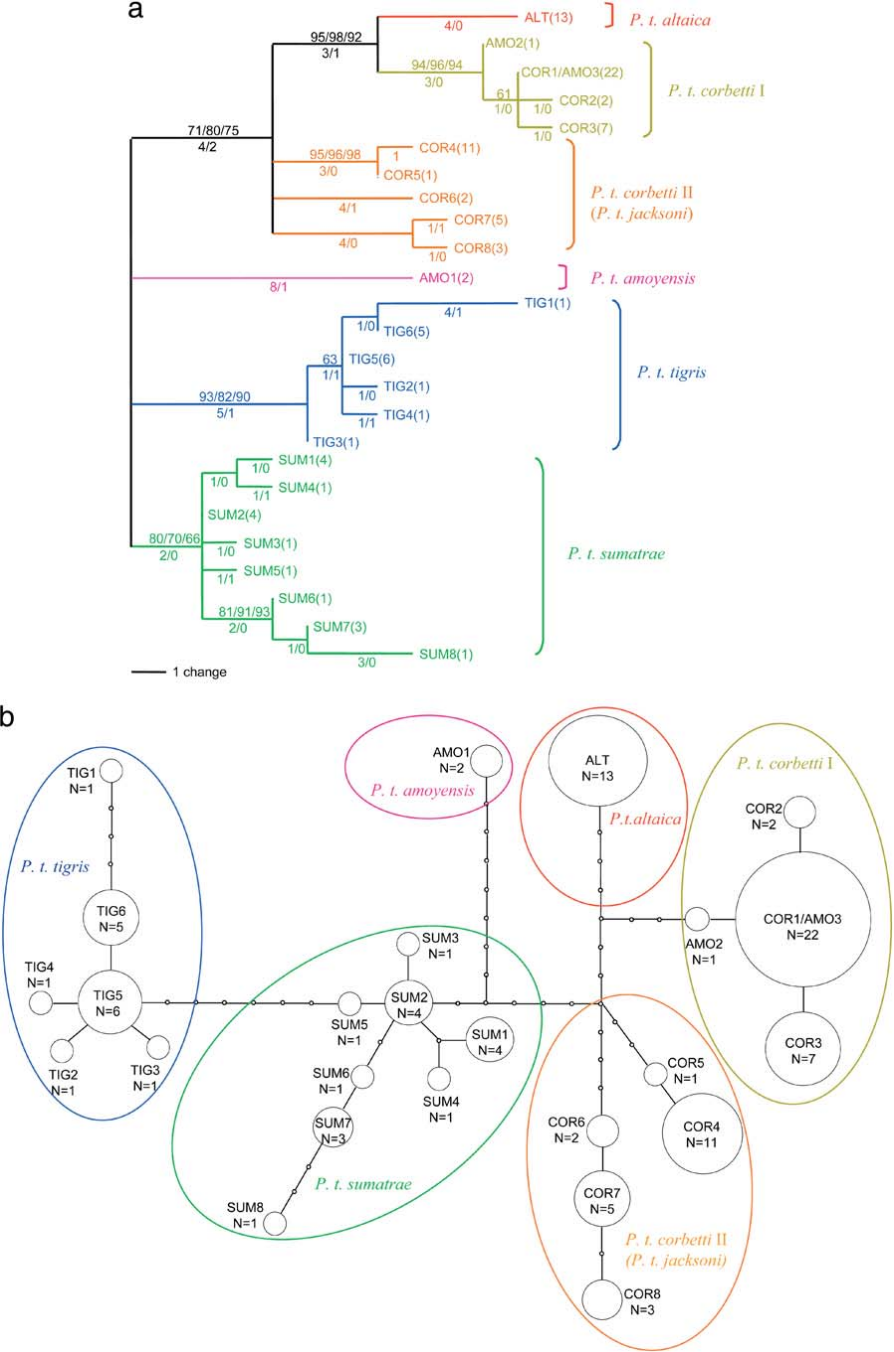
Phylogenetic Relationships among Tigers from mtDNA Haplotypes (A) Phylogenetic relationships based on MP among the tiger mtDNA haplotypes from the combined 4,078 bp mitochondrial sequence (Table 2). Branches of the same color represent haplotypes of the same subspecies. Trees derived from ME and ML analyses have identical topologies. Numbers above branches represent bootstrap support from 100 replicates using the MP method, followed by bootstrap values using the ME-ML analyses (only those over 70% are indicated). Numbers below branches show number of MP steps per number of homoplasies from a strict consensus tree. Numbers in parentheses represent numbers of individuals sharing the same haplotype. MP analysis using heuristic search and tree-bisection-reconnection branch-swapping approach results in two equally most-parsimonious trees and the one resembling the ME and ML trees is shown here (tree length = 60 steps; CI = 0.900). The ME tree is constructed with PAUP using Kimura two-parameter distances (transition to transversion ratio = 2) and NJ algorithm followed by branch-swapping procedure (ME = 0.0142). The ML approach is performed using a TrN (Tamura-Nei) +I (with proportion of invariable sites) model, and all nodes of the ML tree were significant (a consensus of 100 trees, –Ln likelihood = 5987.09). (B) Statistical parsimony network of tiger mtDNA haplotypes based on 4,078 mtDNA sequences constructed using the TCS program (Clement et al. 2000). The area of the circle is approximately proportional to the haplotype frequency, and the length of connecting lines is proportional to the exact nucleotide differences between haplotypes with each unit representing one nucleotide substitution. Missing haplotypes in the network are represented by dots. Haplotype codes and the number of individuals (in parentheses) with each haplotype are shown (see Table 2).

Phylogenetic analyses of the mtDNA haplotypes using maximum parsimony (MP), minimum evolution (ME), and maximum likelihood (ML) approaches produced congruent topologies that defined major geographic partitions (Figure 3A). Eight haplotypes (SUM1 to SUM8) generated from 16 Sumatran tigers *(P. t. sumatrae)* formed a monophyletic group (80% MP, 70% ME, and 66% ML bootstrap support). A second monophyletic cluster of six haplotypes (TIG1 to TIG6) from 15 Bengal tigers *(P. t. tigris)* also received high bootstrap support (93% MP, 82% ME, and 90% ML). The rest of the mainland Asian haplotypes grouped together and partitioned into three distinct geographic groups: (1) a genetically invariant Amur tiger lineage *(P. t. altaica)* represented by a single haplotype in 13 individuals, (2) a northern Indochinese lineage (*P. t. corbetti* I) of individuals originating from south China to the Indochinese countries north of the Isthmus of Kra, and (3) a paraphyletic assembly of haplotypes from tigers from Malayan Peninsula (*P. t. corbetti* II). Support for subdividing the conventional Indochinese subspecies of tigers *P. t. corbetti* into two clusters was high (bootstrap values for *P. t. corbetti* I were 94% MP, 96% ME, and 94% ML). The COR1/AMO3 haplotype, represented by 22 individuals from Vietnam (*n* = 2), Cambodia (*n* = 14), northeast Thailand (*n* = 5), and south China (*n* = 1), was the only haplotype found in two classical subspecies lineages (*P. t. amoyensis* and *P. t. corbetti*) (Table 2).

Voucher samples of five captive tigers collected in China, designated South China subspecies *P. t. amoyensis,* fell into two very distinct phylogenetic origins*.* Two tigers from the Suzhou Zoo (Pti-217 and Pti-218; Table 3) carried the COR1/AMO3 haplotype, and the third (Pti-222) contained haplotype AMO2, which differed by a single nucleotide substitution from COR1/AMO3 (Table 2). The two South China tiger haplotypes grouped phylogenetically with the northern Indochinese *P. t. corbetti* I haplotypes (COR1–COR3) in all phylogenetic analyses (Figure 3A and 3B), and likely indicate that the maternal (mitochondrial) lineages of these tigers derived from individuals from the *P. t. corbetti* I phylogenetic lineages. In contrast, two *P. t. amoyensis* tigers (Pti-219 and Pti-220) from the Chongqing Zoo collection had a haplotype (AMO1) that formed a separate lineage that was ten nucleotide substitutions from its nearest sequence (Sumatran; Figure 3B and Table 2). If affirmed by larger sampling, this lineage would reflect a unique *P. t. amoyensis* genetic haplotype.

A statistical parsimony network of the tiger mtDNA sequences provided additional analytical support for the differentiation of *P. t. sumatrae, P. t. tigris, P. t. altaica, P. t. corbetti* I, *P. t. corbetti* II, and *P. t. amoyensis* (AMO1 only) (Figure 3B). Haplotypes from the same geographic group tended to be interrelated, and intergroup distances among haplotypes were generally larger than branch lengths within each group (1–4 bp). The exceptions were two lineages within the Malayan *P. t. corbetti* II cluster that were separated by 7 bp, which may be a result of the existence of further population substructure or, alternatively, of limited sampling in the region. Each of the six tiger subspecies groups was connected to other groups in close but not exact correspondence to their geographic location. For instance, *P. t. altaica* was the sister taxon to *P. t. corbetti* I which was connected to *P. t. corbetti* II. *P. t. sumatrae* haplotypes were linked to *P. t. tigris* by 7 bp and to *P. t. amoyensis* by 10 bp. Nonetheless, the phylogenetic relationships among the subspecies were not resolved to a robust hierarchy, and therefore were consistent with a contemporaneous divergence of extant phylogeographic lineages.

**Figure 4:**
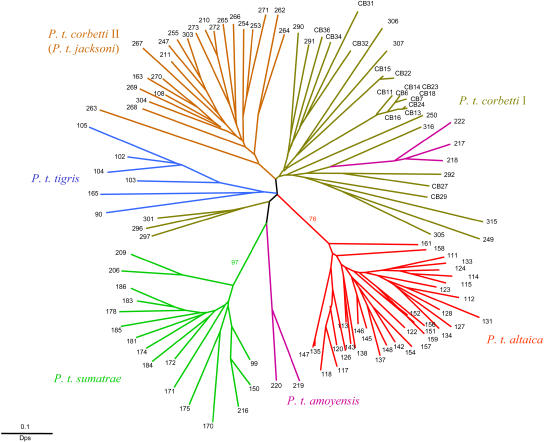
Phylogenetic Relationships among the Individual Tigers from Composite Microsatellite Genotypes of 30 Loci Branches of the same color represent tiger individuals of the same subspecies. The NJ tree, which is based on Dps and Dkf with the (1 – ps/kf) option in MICROSAT (Minch et al. 1995), generated similar topologies, and only the Dps tree is shown here. Numbers are individual Pti codes (Table 3). Bootstrap values over 50% are shown on the divergence node.

Composite genotypes from 30 felid-specific microsatellite loci (Menotti-Raymond et al. 1999) were obtained in 113 tiger samples. Neighbor joining (NJ) analyses of individual tiger genotypes based on the proportion of shared allele (Dps) and kinship coefficient (Dkf) genetic distances produced concordant topologies (Figures 4 and S1) that lend support to the same phylogeographic population subdivisions observed in the mtDNA analysis. Tigers from Sumatra *(P. t. sumatrae)* formed a monophyletic clade with 97% bootstrap support, and Amur tigers *(P. t. altaica)* grouped with 76% bootstrap support. The remaining tiger genotypes partitioned into two weakly supported monophyletic lineages (Indian Subcontinent *P. t. tigris* and Malayan Peninsula *P. t. corbetti* II) and a paraphyletic assemblage of northern Indochinese *P. t. corbetti* I. For example, three individuals from Thailand (Pti-296, Pti-297, and Pti-301) clustered with samples from the India subcontinent, blurring the distinction between *P. t. corbetti* I and *P. t. tigris.* The three South China tigers from the Suzhou Zoo, China, that had clustered with *P. t. corbetti* I by mtDNA (Pti-217, Pti-218, and Pti-222) also associated more closely with *P. t. corbetti* I from northern Indochina by microsatellite analysis (Figure 4). The two distinct (by mtDNA) *P. t. amoyensis* individuals (Pti-219 and Pti-220) from the Chongqing Zoo, China, likewise formed a distinct lineage in the microsatellite analysis (Figure 4).

### Population Subdivision Analysis

To quantify the extent of population differentiation in modern tigers, we evaluated four different geographic subdivision scenarios and compared them on the basis of analysis of molecular variance (AMOVA) with both mtDNA haplotypes and microsatellite genotypes (Table 4). *P. t. amoyensis* individuals (Pti-219 and Pti-220) were excluded in this subdivision analysis due to our small sample size. In our first hypothesis, two groups were considered: the *P. t. sumatrae* island population and all contemporary mainland populations (*P. t. altaica, P. t. corbetti* I, *P. t. corbetti* II, *P. t. tigris)*. This recently proposed model (Cracraft et al. 1998; Kitchener 1999; Kitchener and Dugmore 2000) presumes continuous habitat distribution on the mainland. The second scenario considered tigers as three groups: the Sumatran population *(P. t. sumatrae),* the Amur tigers *(P. t. altaica),* which presently are isolated from other tiger populations by more than their maximum known dispersal distance (Mazak 1996), and a group of the other mainland tigers subspecies. The third hypothesis followed the division of the four traditional subspecies: (1) Amur tigers *(P. t. altaica),* (2) *P. t. corbetti,* including Indochina and part of south China, (3) Bengal tigers *(P. t. tigris),* and (4) Sumatran tigers *(P. t. sumatrae).* The fourth scenario, based on the results of the mtDNA phylogenetic analyses (see Figure 3) and the hypothesis that the Isthmus of Kra may serve as a potential geographic barrier (Kitchener 1999), further subdivided classical *P. t. corbetti* into the northern Indochina region *P. t. corbetti* I and the Malayan Peninsula *P. t. corbetti* II, resulting in five groups. The AMOVA results for each of the four scenarios are presented in Table 4.

**Table 4:**
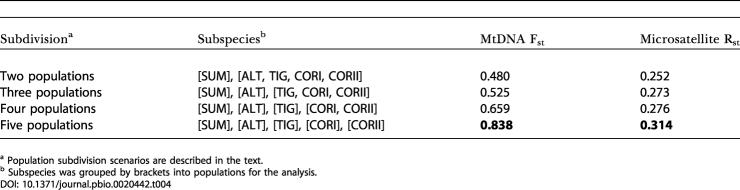
Measures of Geographic Subdivision Based on AMOVA with MtDNA and Microsatellite Data

^a^ Population subdivision scenarios are described in the text

^b^ Subspecies was grouped by brackets into populations for the analysis

For both mitochondrial haplotype and microsatellite data, the five-group scenario yielded the highest F_st_ (for mtDNA, defined as the proportion of total genetic variation that is attributable to genetic differences between populations) and R_st_ (for microsatellites, an F_st_ analogy suited for the stepwise mutation model that applies to microsatellite data) values. Under this model, 31% of the microsatellite variation discriminated between the five groups, while the balance, 69%, occurred within each group. For mtDNA the F_st_ was very high (0.838), indicating that 84% of the variation was partitioned among the different phylogeographic subspecies. Each of the five subspecies showed highly significant population genetic differentiation (*p* < 0.0001) by pairwise F_st_ and R_st_ with 10,000 permutations (Table 5). The contrast between the mtDNA and microsatellite genetic variation probably reflects the difference in the effective population size assessed by these two different markers and/or, to some extent, the intersexual differences in dispersal.

**Table 5:**
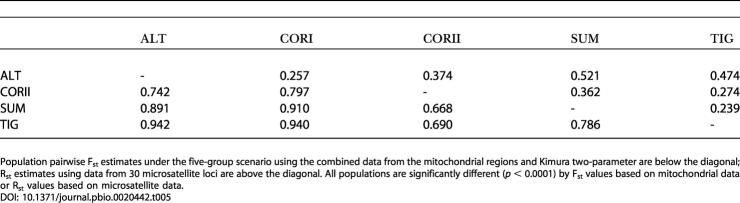
Measures of Pairwise Comparisons in Tigers Based on AMOVA with mtDNA and Microsatellite Data

Population pairwise F_st_ estimates under the five-group scenario using the combined data from the mitochondrial regions and Kimura two-parameter are below the diagonal; R_st_ estimates using data from 30 microsatellite loci are above the diagonal. All populations are significantly different (*p* < 0.0001) by F_st_ values based on mitochondrial data or R_st_ values based on microsatellite data

An alternative analysis of the combined microsatellite and mitochondrial haplotype data using a Bayesian approach (Figure S2 and Table S1) as implemented in the program STRUCTURE (Pritchard et al. 2000) supported the partitioning of *P. t. altaica, P. t. sumatrae, P. t. tigris,* and *P. t. corbetti* II, but further split the 33 *P. t. corbetti* I individuals into three distinctive population groups: (1) four tigers from China and Vietnam; (2) nine tigers from Cambodia; and (3) 20 tigers from Cambodia and northern Thailand (*K* = 7, *Pr*[*K*] = 0.993). In this scenario, most individuals were assigned to a cluster with high probability (*q* > 0.90), indicating very low level of gene flow between the groups. However, because this additional substructure within *P. t. corbetti* I had little geographic or ecological basis, and because AMOVA analysis based on this population subdivision resulted in lower F_st_ and R_st_ values than that in the five-group scenario (unpublished data), the distinction was not considered to be a consistent basis for subspecies classification and may reflect additional population differentiation within a subspecies.

### Genetic Variation in Tigers

Quantitative estimates of mtDNA diversity in tigers with comparable estimates from selected felid species demonstrated that overall, tigers had moderate levels of mtDNA diversity (Table 6), substantially less than leopards *(P. pardus)* (Uphyrkina et al. 2001), Geoffroy's cat *(Oncifelis geoffroyi),* Pampas cat *(O. colocolo),* or tigrina *(Leopardus tigrinus)* (Johnson et al. 1999), but comparable to pumas *(Puma concolor)* (Culver et al. 2000) in percent variable sites, mean pairwise distance among individuals, and average nucleotide diversity. Four tiger subspecies (*P. t. tigris, P. t. sumatrae, P. t. corbetti* I, and *P. t. corbetti* II) showed moderate nucleotide diversity (π), ranging from 0.0001 to 0.0070 (Table 6). The *P. t. altaia* sampling of 13 individuals showed no mtDNA haplotype variation. Of the five individuals originally designated as *P. t. amoyensis,* three were genetically indistinguishable from *P. t. corbetti* I, resulting in an inadequate sample size for a meaningful estimation of population variation.

**Table 6:**
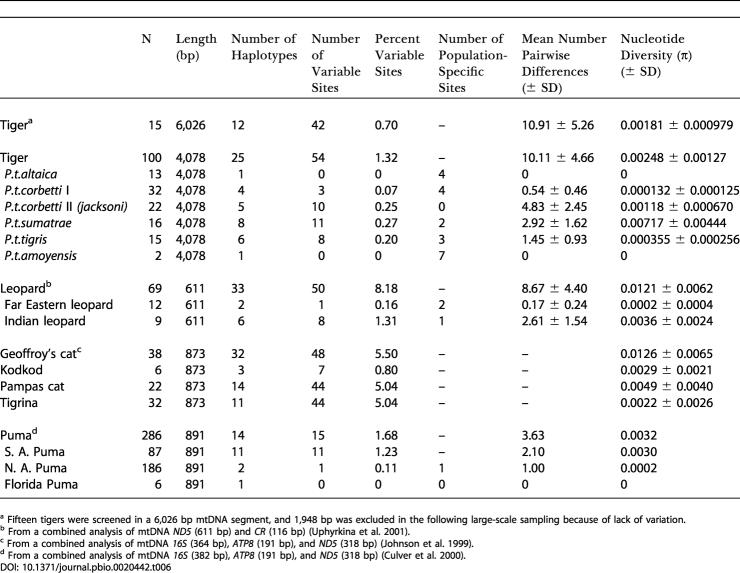
Estimates of Molecular Genetic Variation from Combined MtDNA Sequences (4,078 bp)

^a^ Fifteen tigers were screened in a 6,026 bp mtDNA segment, and 1,948 bp was excluded in the following large-scale sampling because of lack of variation

^b^ From a combined analysis of mtDNA *ND5* (611 bp) and *CR* (116 bp) (Uphyrkina et al. 2001)

^c^ From a combined analysis of mtDNA *16S* (364 bp), *ATP8* (191 bp), and *ND5* (318 bp) (Johnson et al. 1999)

^d^ From a combined analysis of mtDNA *16S* (382 bp), *ATP8* (191 bp), and *ND5* (318 bp) (Culver et al. 2000)

Parameters of microsatellite variation have been shown to provide sensitive measures of historic demographic perturbations in felid and other species (Driscoll et al. 2002). Estimates of heterozygosity, average numbers of allele per locus, microsatellite variance in allele size, and allele size range in tigers were comparable to other felid species such as jaguar, leopard, puma, lions, and cheetahs across the same microsatellite loci (*n* = 17) (Table 7). After Bonferroni correction, eight of the 30 loci were significantly out of Hardy-Weinberg equilibrium in *P. t. corbetti* I (*p* < 0.00167), possibly reflecting further population subdivision in this region. Expected heterozygosity in tigers ranged from 0.456 in *P. t. altaica* to 0.670 in *P. t. corbetti* I (Table 7). Average microsatellite variance was highest in *P. t. tigris* (4.94) and *P. t. corbetti* I (3.58) and lowest in *P. t. altaica* (1.93).

**Table 7:**
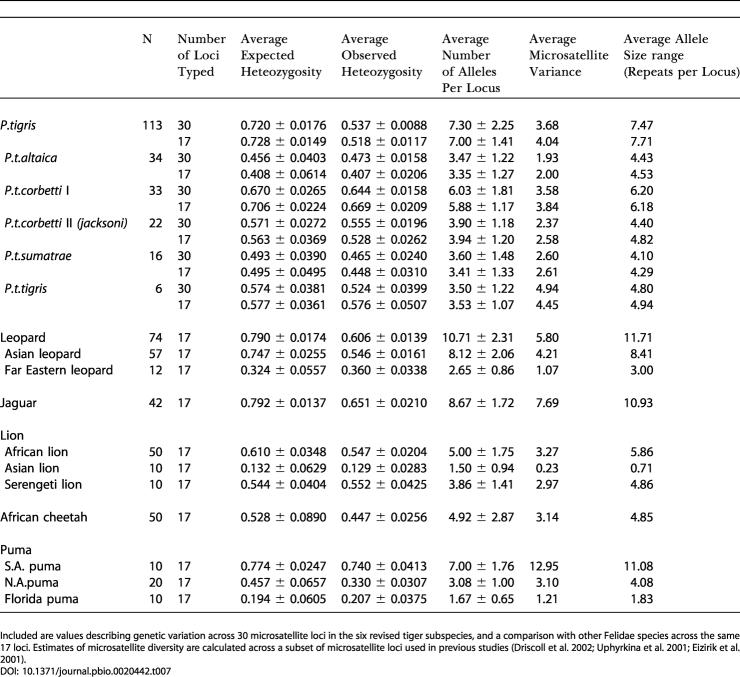
Genetic Variation across 30 Microsatellite Loci in Tiger Subspecies

Included are values describing genetic variation across 30 microsatellite loci in the six revised tiger subspecies, and a comparison with other Felidae species across the same 17 loci. Estimates of microsatellite diversity are calculated across a subset of microsatellite loci used in previous studies (Driscoll et al. 2002; Uphyrkina et al. 2001; Eizirik et al. 2001)

All six phylogeographic subspecies groups showed population-specific alleles that tended to represent the extreme sizes of allele distributions (Table 8). Of the 49 private alleles, 26 were either the largest or smallest size class among all tigers, and 38 were either the smallest or the largest for a specific subspecies, thus supporting a recent derivation. Frequencies of such private alleles were low in each population, from 1.5% of total allele numbers in *P. t. amoyensis* to 14.6% in *P. t. corbetti* I (Table 8)*.* In addition, *P. t. corbetti* I had the highest average number of alleles per locus, the highest average allele size range per locus, and the most continuous and heterogeneous allele size distribution among all subspecies groups.

**Table 8:**
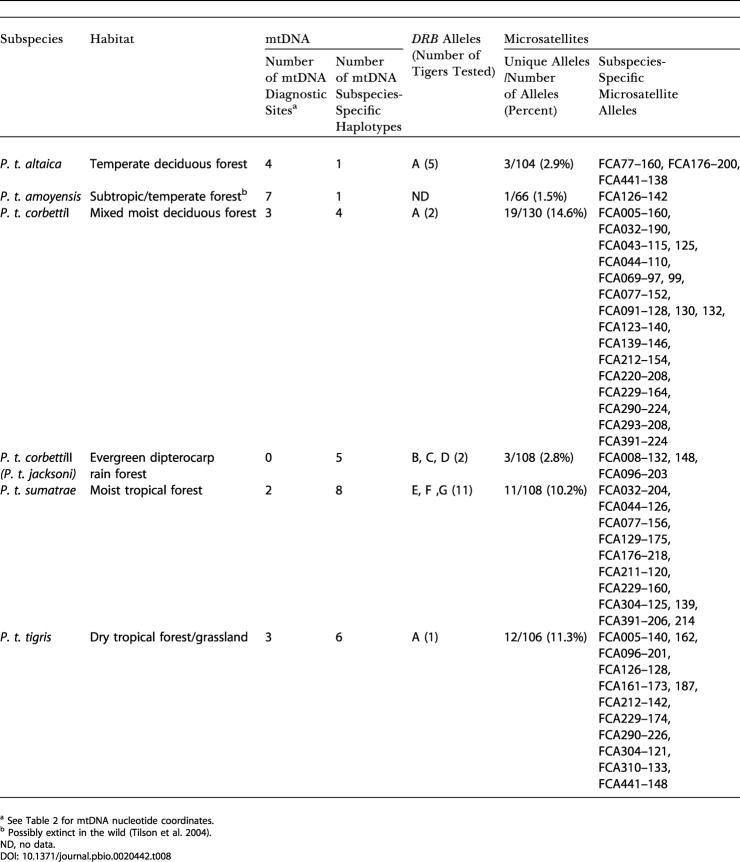
Diagnostic Characters and Habitat of the Six Phylogeographic Tiger Groups or Subspecies

^a^ See Table 2 for mtDNA nucleotide coordinates

^b^ Possibly extinct in the wild (Tilson et al. 2004)

ND, no data

### Major Histocompatibility Complex—*DRB* Gene Variation

The most polymorphic gene complex in all mammals is the MHC. This critical region for immunological recognition of infectious agents has 147 genes in the domestic cat, including three functional class II *DRB* genes on chromosome B3 (Yuhki et al. 2003). *DRB* gene homologs were amplified from DNA extracted from 21 tigers and screened for sequence diversity using single strand conformational polymorphism (SSCP). There were a total of seven electrophoretic allele variants (A–G). This is a relatively low MHC-*DR* diversity compared to human and domestic cat, which possess 126 and 63 *DRB* alleles, respectively, for the same gene segment in samplings of 251 humans and 37 cats, respectively (Yuhki and O'Brien 1997; Bodmer et al. 1999). Despite this reduced *DRB* variation among tigers, there was detectable population differentiation. Three mainland subspecies *P. t. tigris* (*n* = 1), *P. t. altaica* (*n* = 5), and *P. t. corbetti* I (*n* = 2) were genetically identical for *DRB*-A allele sequence. Three additional *DRB* alleles (B, C, and D) were found only in *P. t. corbetti* II (*n* = 2), while three others (E, F, and G) were unique to *P. t. sumatrae* (*n* = 11) (Tables 3 and 8) (Wentzel et al. 1999).

### Estimation of the Coalescence Time of Genetic Variations in Tigers

The mtDNA sequence divergences in a combined data set of 3,217 bp, of which homologous sequences from the tiger and leopard were both determined (see Materials and Methods), were used to estimate coalescence time for extant tiger mtDNA lineages and its 95% confidence interval (CI: ± two standard errors) based on a linearized tree method (Takezaki et al. 1995). Neither the two-cluster nor the branch-length molecular clock test revealed significant rate heterogeneity among tiger sequences (confidence probability less than 95%), suggesting that the divergence of the mtDNA sequences were compatible with a molecular clock hypothesis. Thus, all sequences were used to construct a linearized tree using the NJ tree algorithm with Kimura two-parameter distances. Assuming a divergence time for leopards and tigers of 2 MY, there were an estimated 2.29 × 10^–8^ substitutions per site per y, or one substitution every 14,000 y in the segment examined. According to this rate, the estimated coalescence time of mtDNA variation for extant tiger lineages was 72,000 y (95% CI = 39,000–104,000 y). An older fossil record calibration of 3 MY for the separation of leopards and tigers produced a rate of 1.53 × 10^–8^ substitutions per site per y, or one substitution every 20,000 y. According to this substitution rate, mtDNA diversity of modern tigers originated about 108,000 y (95% CI = 59,000–157,000 y) ago. Based on either calibration, the Amur tigers probably experienced a genetic reduction or founder event more recently (less than 20,000 y), as no variation was detected within the population.

The estimate of microsatellite variance in average allele repeat-size can also be used as a surrogate for evolutionary time based on the rate of allele range reconstitution subsequent to a severe founder effect (Driscoll et al. 2002). Using a standard curve for the relationship of microsatellite variance to elapsed time (see Figure 4 in Driscoll et al. [2002]), the variance for all tigers converged to 19,000 y ago. The age of different subspecies, based on populations for which we had an adequate sample size (*n* > 15), ranged from 9,900 y in Amur tigers *P. t. altaica* to 18,437 y in northern Indochinese tigers *P. t. corbetti* I.

We estimated the historic population size required to sustain the level of mitochondrial genetic variation under the assumption of neutrality of substitution and mutation-drift equilibrium (Kimura 1955; Nei 1987), where the population parameter θ = 2*N*_e_ *μT,* and *N*_e_ is the long-term effective female population size, *μ* the substitution rate per site per year, and *T* the generation time. From a coalescent-based simulation of the mitochondrial sequences, the average estimate of θ was 0.00255 per nucleotide site, with a 95% CI from 0.00147 to 0.00417. With the substitution rate calibrated from this study (1.91 × 10^–8^ bp^–1^ y^–1^) and an average generation time of 5 y for tigers (Smith and McDougal 1991), the historical effective population size is an estimated 13,350 females (95% CI = 7,700–21,830).

## Discussion

Overall, tigers displayed moderate levels of molecular genetic variation in mtDNA and *DRB* sequences compared with other mammalian species, consistent with previous allozyme studies (O'Brien et al. 1987). There was a variable site every 75 bp, with 54 sites in the more variable 4-kb segment and one variable site every 112 bp in the larger 6,026-bp segment (see Materials and Methods). This value was less than what was observed in leopards in a smaller portion of mtDNA (one variable site every 15 bp in 727 bp of the gene encoding NADH dehydrogenase subunit 5, called *ND5,* and that for the control region, called *CR;* 34 haplotypes were found) (Uphyrkina et al. 2001). MHC class-II *DRB* gene variation was also low relative to human and domestic cat (Yuhki and O'Brien 1997; Bodmer et al. 1999). By contrast, estimates of tiger microsatellite variability were more similar to those of other felid species (Table 7) (Culver et al. 2000; Eizirik et al. 2001; Uphyrkina et al. 2001; Driscoll et al. 2002).

The oldest tiger fossils, around two million y (MY) old, are from northern China and Java (Hemmer 1987). By the late Pliocene and early Pleistocene tigers were widely distributed in eastern Asia. However, Pleistocene glacial and interglacial fluctuations and other geological events probably caused repeated geographic restrictions and expansions (Hemmer 1987; Kitchener 1999; Kitchener and Dugmore 2000). We estimated the most recent common ancestor for tiger mtDNA haplotypes was 72,000–108,000 y ago, with a lower and upper bound of 39,000 y and 157,000 y, respectively. This estimate is much earlier than that derived for the leopard, which is considered to have originated in Africa 470,000–825,000 y ago and to have arrived in Asia 170,000–300,000 y ago (Uphyrkina et al. 2001). Likewise, extant jaguar *(Panthera onca)* lineages diverged approximately 280,000–510,000 y ago (Eizirik et al. 2001). Our coalescence estimate for tigers corresponds roughly with the catastrophic eruption of Toba in Sumatra around 73,500 y ago (Rampino and Self 1992), which has been linked to the Late Pleistocene bottleneck in human evolution (Ambrose 1998) and to a major northward dispersal event in the Asian elephants (Fleischer et al. 2001).

Based on the subspecies definition of O'Brien and Mayr (1991) and Avise and Ball (1990), our data suggest that there are at least five and possibly six tiger subspecies: Amur tigers *(P. t. altaica);* northern Indochinese tigers (*P. t. corbetti* I); southern Indochinese tigers (*P. t. corbetti* II), which are confined to the Malayan Peninsula; Sumatran tigers *(P. t. sumatrae);* Bengal tigers *(P. t. tigris);* and, if its uniqueness is affirmed by more extensive sampling, South China tiger *(P. t. amoyensis)*. These conclusions are based on significant genetic structure among tigers from these different geographic regions with the MHC, mtDNA, and microsatellite data, and extremely limited gene flow as shown by disjunct distributions of genetic variation (unique mtDNA haplotypes and microsatellite alleles) and the high mtDNA F_st_ and microsatellite R_st_ values. In addition, each subspecies has an allopatric geographical distribution (see Figure 1) and differential natural history (Kitchener 1999; Seidensticker et al. 1999).

The hypothesis that tiger population structure reflects recent (less than 10,000 y ago), human-induced population fragmentation and random lineage loss from a single panmictic population is not supported by the strong geographical partitioning of the mitochondrial lineages or by differences in measures of nucleotide diversity within each subspecies. Mismatch analysis (Rogers and Harpending 1992) of pairwise differences among all tiger mtDNA haplotypes also revealed a multimodal distribution significantly different from a Poisson expectation, indicating the existence of several highly divergent populations (unpublished data). It is plausible that tiger populations (subspecies) differentiated through the combined effects of genetic drift in isolated populations and local adaptation to rapidly changing habitats across the tiger range during the Holocene (Lister 2004). For example, Sumatran tigers currently occupy tropical moist forests, and Bengal tigers range from tropical dry forests, terai forests, and tall grasslands to the Himalayan foothills. However, we cannot rule out the possibility that some of the current population subdivision, particularly in the case of the divergence of *P. t. altaica* and *P. t. amoyensis/P. t. corbetti* I, could be related to the disruption of an isolation-by-distance pattern caused by the recent extinction of intermediate populations; this hypothesis can be tested only when a larger geographic sampling is available.

The differences in molecular genetic patterns among the six hypothesized subspecies are dramatic (Table 8). Further, the results lend support to the hypothesis that the Pleistocene centrum of tiger radiation is located within northern Indochina and southern China. Modern *P. t. corbetti* I has a large number of mtDNA diagnostic sites (three), the largest number of unique microsatellite alleles (19 out of 130), and the highest overall microsatellite diversity (Tables 7 and 8). In addition, no microsatellite allele at any locus occurred with a frequency higher than 81%. The observed allele size distribution in *P. t. corbetti* I was generally continuous for most loci (there were fewer allele size gaps compared to other subspecies), evidence of a fairly stable demographic history, and alleles found in the other subspecies were almost always a subset of those found in *P. t. corbetti* I.

Additional sampling of modern and/or historic samples could reveal additional structure (putative subspecies) in the *P. t. corbetti* I region (see Figure 1), as there were several microsatellite loci out of Hardy-Weinberg equilibrium, and the Bayesian population structure analysis identified possible substructure within *P. t. corbetti* I (Figure S2). The ultimate classification of tigers of the southern China and northern Indochina region is further complicated by the poor definition of the geographic boundary between *P. t. corbetti* I and *P. t. amoyensis,* and because the South China tiger subspecies is represented only by captive-born animals of imprecise origin. One of the two phylogenetic lineages in this captive population (Pti-217, Pti-218, and Pti-222) was indistinguishable from northern Indochinese tigers (see Figures 3 and 4), perhaps as a consequence of introgression of the northern Indochinese tigers into the Chinese captive population or a more-northern distribution of the Indochinese tigers than had previously been recognized. A comprehensive morphological and genetic assessment of the captive population (around 50 individuals) (Tilson et al. 2004), of historic samples, and of additional wild tigers from southern China, in the context of subspecies patterns seen here would be useful to resolve remaining uncertainties and to inform in situ and ex situ management strategies.

By contrast, the other subspecies delineations are better defined. To the north, Amur tigers, presently an isolated population of fewer than 500 individuals, are confined almost entirely to the Russian Far East (Matyushkin et al. 1999). They display low genetic diversity in comparison to other subspecies, with a single mtDNA haplotype that is likely derived from *P. t. corbetti* I Indochinese tigers (Figure 3A). The Amur tiger genetic variability may have been reduced during a post-ice age colonization of the region around 9,000 y ago and/or during the early 20th century when an estimated 20–30 tigers survived intense human persecution (Kaplanov 1948). In Indochina, the genetic distinction between *P. t. corbetti* I and *P. t. corbetti* II (pairwise mtDNA F_st_ = 0.797 and microsatellite R_st_ = 0.225, *p* < 0.0001; *P. t. corbetti* II is characterized by three unique microsatellite alleles and five subspecies-specific mtDNA haplotypes [Table 8]) supports the hypothesis that the Isthmus of Kra has been an ecological barrier restricting gene flow between tigers in Malaya Peninsula and mainland Southeast Asia. Previous biogeography studies have placed numerous species and subspecies boundaries of mammals (Corbett and Hill 1993; Tosi et al. 2002), birds (Hughes et al. 2003), and plants (Woodruff 2003) near the Isthmus of Kra, making it a significant biogeographical transition between Indochina and Sundaic regions.

The isolation of Sumatran tigers from mainland populations is supported by multiple unique characters, including two diagnostic mtDNA nucleotide sites, eight mtDNA haplotypes, and 11 (of 108) unique microsatellite alleles (Table 8). Cracraft et al. (1998) and Hendrickson et al. (2000) also described genetic variation distinguishing Sumatran tigers from other tiger subspecies. The relatively high genetic variability and phylogenetic distinctiveness of Sumatran tigers suggest a historically large effective population size followed by highly restricted gene flow between the island and other populations.

The Bengal tigers are defined by three distinct mitochondrial nucleotide sites and 12 unique microsatellite alleles. The pattern of genetic variation in the Bengal tiger corresponds to the premise that tigers arrived in India approximately 12,000 y ago (Kitchener and Dugmore 2000). This recent history of tigers in the Indian subcontinent is consistent with the lack of tiger fossils from India prior to the late Pleistocene and the absence of tigers from Sri Lanka, which was separated from the subcontinent by rising sea levels in the early Holocene. Similar biogeographical boundaries to those separating the six tiger subspecies have been proposed in other species including leopard (Uphyrkina et al. 2001), Asian elephant (Fleischer et al. 2001), and rodents (Gorog et al. 2004), but warrant further study to determine their importance as recent barriers to gene flow for large mammals in Asia.

Our results have several implications for tiger conservation. Management strategies for the tiger, both in situ and ex situ, have been historically influenced by perceptions of its geographical variation and subspecific taxonomy (Maguire and Lacy 1990; Seidensticker et al. 1999), and several captive tiger breeding programs have attempted to maintain purebred lines (Foose 1987; Maguire and Lacy 1990). Our data suggest, however, that while supporting and refining most existing (and extant) tiger subspecies designations, there is additional substructure within some subspecies that should be considered when formulating management strategies for captive animals or when considering the maintenance of sufficiently large and interconnected wild populations. Specifically, the distinctiveness of tigers from Malayan Peninsula is comparable to differences among other recognized and separately managed subspecies. To be consistent, the Malayan subspecies should also be managed as such unless inbreeding depression has become an issue due to declined genetic variability. Since the current type specimen for *P. t. corbetti* is located in northern Vietnam (Mazak 1968), and no prior name has been given to the southern populations, we propose the newly defined tiger subspecies from Malayan Peninsula be designated *P. t. jacksoni,* to honor the contributions of Peter Jackson, the former Chair of the the World Conservation Union (IUCN) Cat Specialist Group, who tirelessly labored for more than 40 y on behalf of tiger conservation. We designate the type specimen of the Malayan tigers to Pti-163 from the Zoo Melaka, Malaysia, and the taxonomic diagnosis will be described elsewhere. The present status of tigers from northern Indochina and from Malayan Peninsula is uncertain, urging more extensive study and conservation.

Our results also show that, although modern tigers have a relatively young history, ecological, demographic, and biogeographic factors have led to recognizable subdivisions among otherwise closely related populations. We therefore might expect that more extensive geographic sampling would reveal additional phylogenetic divisions among populations, especially in the Indian Subcontinent and the Indochina bioregions, or alternatively, would blur the apparent phylogenetic subdivisions and reveal a clinal distribution of genetic variation across different subspecies. Further sampling of modern and historic specimens will also help clarify whether the patterns we have observed are attributable to the recent substantial population decline throughout the range in tigers, or whether the observed differentiations among tigers occurred earlier.

## Materials and Methods

### 

#### Samples.

A total of 134 tiger individuals were sampled throughout the distribution range (see Figure 1 and Table 3). Of these, 100 were verified as either wild-born from a specific geographic locale or captive-born from geographically verified wild-born parents. An additional 34 individuals were of reasonably certain geographic origin and were used to complement estimated levels of molecular genetic variation in tigers. Individuals were labeled with traditional subspecies classifications based on their geographical origin following Mazak (1996). Genomic DNA from blood or primary skin fibroblast cell culture was isolated using a standard proteinase K digestion and phenol-chloroform extraction procedure (Sambrook et al. 1989). DNA was isolated from dry skin using guanidine thiocyanate (Boom et al. 1990) and silica-based purification (Hoss and Paabo 1993). DNA from hair was obtained by a modification of the previously described chelex method (Higuchi et al. 1988). Analysis of historical samples was carried out with strict precautions at an isolated laboratory specializing in work with ancient DNA and was independently repeated to exclude possible contamination from any high-copy DNA source (Hofreiter et al. 2001).

#### Mitochondrial DNA analysis.

Analyses of mtDNA in tigers and in other *Panthera* species is complicated by the presence of a large 12.8 kb nuclear mtDNA fragment that transposed to chromosome F2 in an ancestral *Panthera* species approximately 3 MYA (Johnson et al. 1996; Lopez et al. 1996; Cracraft et al. 1998; J. H. Kim, A. Antunes, S.-J. Luo, J. Menninger, W. G. Nash, et al., personal communication). Although the Numt and Cymt DNA sequences have diverged, primers designed from conserved regions often coamplify both copies. Fifteen Cymt-specific primer sets (see Figure 2 and Table 1) were designed on the basis of sequence differences from the alignments of the complete tiger Numt and the homologous 12.8-kb Cymt sequences (J. H. Kim, A. Antunes, S.-J. Luo, J. Menninger, W. G. Nash, et al., personal communication). These Cymt primers amplified a total of 6,026 bp of sequence, spanning ten mitochondrial gene segments, including NADH dehydrogenase subunits 1, 2, 5, and 6 (*ND1*, *ND2*, *ND5,* and *ND6*), cytochrome B*(CytB),* control region*(CR),* 12S rRNA*(12S),* cytochrome C oxidase subunits I and II*(COI* and *COII),* and ATPase8 *(ATP8)* (see Figure 2). The primer sets were tested in 15 individuals representing tigers from all five traditional subspecies. Five segments that revealed no variation in the pilot screening were excluded from further analysis (see Figure 2).

PCR products were amplified from 50 ng of genomic DNA in a 25 μL reaction system containing 2.0 mM MgCl_2_, 1.0 mM dNTPs, 0.25 units of AmpliTaq Gold DNA polymerase (Applied Biosystems, Foster City, California, United States), and 1× PCR buffer II; the amplification protocol was: denaturation 10 min at 95 °C, a touch-down cycle of 95 °C for 30 s, 52 °C for 30 s decreased by 1 °C in the next cycle for 10 cycles, 72 °C for 45 s, then 35 amplification cycles of 95 °C for 30 s, 52 °C for 30 s, and 72 °C for 45 s, followed by an extension of 10 min at 72 °C. PCR products were purified using Microcon PCR filters (Millipore, Billerica, Massachusetts, United States) and were directly sequenced in both directions using BigDye Terminator kits (Applied Biosystems) and run on an ABI 377 sequencing apparatus. Sequences were inspected using SEQUENCHER (Gene Codes, Ann Arbor, Michigan, United States), unambiguously aligned using Clustal-X (Thompson et al. 1997), and visually inspected. Sequences for each mtDNA fragment were combined for a total evidence approach. Sequences were deposited in GenBank.

Phylogenetic relationships among mtDNA haplotypes were assessed using three approaches implemented in PAUP (Swofford 2001). An MP analysis was conducted using a heuristic search, with random additions of taxa and tree-bisection-reconnection branch swapping. The ME heuristic search approach consisted of NJ trees constructed from Kimura two-parameter distances followed by a branch-swapping procedure. ML analysis was done using the TrN (Tamura-Nei) +I (with proportion of invariable sites) model with the proportion of invariable sites set to 0.93, and the rate among sites equal, as estimated using MODELTEST 3.06 (Posada and Crandall 1998). The reliability of the nodes in each of the analyses was assessed by 100 bootstrap iterations. A statistical parsimony network was constructed using TCS 1.13 (Clement et al. 2000) to infer phylogeographic and potential ancestor-descendent relationships among haplotypes. Measures of population genetic variation, such as mean number of pairwise differences, gene diversity, and nucleotide diversity were estimated using ARLEQUIN 2.0 (Schneider et al. 2000). The extent of geographic subdivision among populations was assessed by F_st_ values (with Kimura two-parameter distance) using AMOVA as implemented in ARLEQUIN 2.0. Statistical significance was tested using 10,000 permutations.

The approximate coalescence time of tigers was estimated with a linearized tree method as implemented in the program LINTRE (Takezaki et al. 1995). This program constructs linearized NJ trees reestimating branch lengths under the molecular clock assumption and incorporates two tests for the assumption (Takezaki et al. 1995). The mtDNA sequence divergence was based on the standard equation *H* = 2μ*T*, where *H* was the branch height in the linearized tree correlated to the average pairwise distance among haplotypes, μ the substitution rate, and *T* the divergence time. Since there was no comparable sequence from other *Panthera* species available, we generated from PCR and GenBank a chimera-homologous sequence of 3.2 kb (including fragments from mitochondrial genes *ND1, ND2, ND5, ND6, CytB, 12S,* and *COI*) from three leopard individuals. The divergence time between leopard and tiger was used as a calibration point, and two fossil dates were chosen. Two million y was a commonly used lower bound for the *Panthera* lineage radiation and the date for the earliest reported tiger in the fossil record (Hemmer 1987; O'Brien et al. 1987; Wayne et al. 1991). An earlier record of 3 million y was also chosen because leopard fossils have been reported from this time period (Turner and Anton 1997). Domestic cat *(Felis catus)* was used as an outgroup. Coalescent-based simulation of the population parameter θ estimation and its 95% CI was conducted in DnaSP 4.0 (Rozas et al. 2003) with 1,000 replicates, given that the mutations along the lineages followed a Poisson distribution.

#### Microsatellite analysis

Thirty polymorphic microsatellite loci (FCA005, FCA008, FCA032, FCA043, FCA044, FCA069, FCA077, FCA090, FCA091, FCA094, FCA096, FCA105, FCA123, FCA126, FCA129, FCA139, FCA161, FCA176, FCA201, FCA211, FCA212, FCA220, FCA229, FCA242, FCA290, FCA293, FCA304, FCA310, FCA391, and FCA441) originally designed in the domestic cat *(F. catus)* (Menotti-Raymond et al. 1999) were amplified by PCR using fluorescently labeled primers under previously published conditions (Menotti-Raymond et al. 1999). Two loci (FCA391 and FCA441) were tetranucleotide repeats, and the others were dinucleotides. All loci have been mapped in the domestic cat and located on 11 of the 19 chromosomes (Menotti-Raymond et al. 1999; Menotti-Raymond et al. 2003). These microsatellites were in different linkage groups or at least 12 centimorgans apart in the domestic cat, except for FCA 211 and FCA 212 (4 centimorgans), and were likely to be in linkage equilibrium. The dye-labeled PCR products of the 30 microsatellite primer sets were pooled and diluted based on size range and fluorescent dye so that 2–4 loci could be multiplexed and subsequently analyzed by electrophoresis in an ABI 377 automated sequencer (Applied Biosystems). Patterns were scored and analyzed using GENESCAN 2.1 and GENOTYPER 2.5 software. Of 134 tiger samples, 113 were included in the microsatellite analysis. DNA samples from pelt or hair for which fewer than 20 of the loci amplified successfully were excluded from the analysis.

Tests for genotypic linkage disequilibrium and deviations from Hardy-Weinberg equilibrium for each locus in each population were performed using GENEPOP web version of 3.1c (http://wbiomed.curtin.edu.au/genepop/) (Raymond and Rosset 1995). Measures of microsatellite genetic variation in terms of average observed heterozygosity and expected heterozygosity, average number of alleles per locus, average allele size range per locus, number of unique alleles, and average variance were estimated with MICROSAT (Minch et al. 1995). Pairwise genetic distances among individual tigers were estimated based on Dps and Dkf with the [1 – ps/kf] option in MICROSAT and were used to construct NJ phylogenetic trees with the program NEIGHBOR in the PHYLIP 3.5 package (Felsenstein 1989). Assessments of different geographic subdivision scenarios and population pairwise comparisons (using R_st_, sum of square size differences) were derived from ARLEQUIN 2.0. The statistical significance of R_st_ values, sum of squared size differences, was tested with 10,000 permutations as implemented in ARLEQUIN. A Bayesian clustering method implemented in the program STRUCTURE (Pritchard et al. 2000) was used to infer population structure based upon multilocus microsatellite genotype and sequence data. MtDNA was treated as a single haploid locus, and each observed haplotype was coded with a unique integer (e.g., 1, 2) for the first allele and the missing data symbol (e.g., -9) for the second. We calculated the probability of individual assignments to population clusters *(K)* without prior information of the origin of individuals. A series of tests was conducted using different numbers of population clusters to guide an empirical estimate of the number of identifiable populations, assuming an admixture model with correlated allele frequencies and with burn-in and replication values set at 50,000 and 10^6^, respectively. Each test yielded a log likelihood value of the data (Ln probability), the highest of which would indicate which test was closest to the actual number of genetically distinct populations. These tests also provided an alpha value, the measure of admixed individuals in the data set.

#### Class II MHC.

Allele variation in the nuclear MHC class II *DRB* gene was assessed in five Amur, two northern Indochinese, two Malayan, 11 Sumatran, and one Bengal tiger. Conserved PCR primers designed from the human *DRB* sequence were used to amplify homologous *DRB* sequences (of around 238 bp) in 21 tiger voucher DNAs with primers 61a (5′- CCGCTGCACTGTGAAGCT-3′) and 219a (5′- CCACACAGCACGTTTCTT-3′). Products were screened for polymorphisms using SSCP, a method that detects single-basepair substitutions in 100–300-bp DNA fragments. For SSCP, PCR products were mixed with a solution of 120 μl of formamide, 20 μl of TAMRA (Applied Biosystems) lane standard, and 20 μl of a blue dextran loading dye (from a stock solution of 50 mg/ml with 25 mM EDTA), then denatured at 95 °C for 3 min. The electrophoresis ran at 2,000 volts, 400 amps, and 25 watts in 1× TBE buffer through a 6% denaturing polyacrylamide gel (19.5:1 acrylamide:bis). The SSCP fragments were visualized by autoradiography, and alleles were scored by eye (Yuhki and O'Brien 1997).

## Supporting Information

Figure S1 Phylogenetic Relationships among the Individual Tigers from Composite Microsatellite Genotypes of 30 Loci Branches of the same color represent tiger individuals of the same classically named subspecies. NJ tree constructed based on kinship coefficient (Dkf) with the (1 – kf) option in MICROSAT (Minch et al. 1995). Numbers are individual Pti codes (Table 3). Bootstrap values over 50% are shown on divergence nodes.
(108 KB DOC).

Figure S2 Bayesian Population Structure Analysis of 111 Tigers Data obtained from microsatellite genotype and mitochondrial haplotype data were analyzed using STRUCTURE (Pritchard et al. 2000). Simulations were set at 50,000 burn-in period followed by 10^6^ replicates. Each individual is represented by a thin vertical bar, which is partitioned into *K* colored segments that represent the individual affiliation to each of *K* clusters. Here shows the population structure when *K* = 7, which produced the highest probability among other choices of *K*. Three STRUCTURE runs produced almost identical individual affiliation.
(62 KB DOC).

Table S1 Bayesian Clustering Analyses for Tiger Microsatellite and Mitochondrial Data (59 KB DOC).

### Accession Numbers

The GenBank (http://www.ncbi.nlm.nih.gov/) accession numbers of the mtDNA fragments discussed in this paper are AY736559–AY736808.

## Abbreviations

AMOVA: analysis of molecular variance
CI: confidence interval
Cymt: cytoplasmic mitochondrial
Dkf: kinship coefficient
Dps: proportion of shared alleles
ME: minimum evolution
MHC: major histocompatibility complex
ML: maximum likelihood
MP: maximum parsimony
mtDNA: mitochondrial DNA
MY(A): million years (ago)
NJ: neighbor joining
Numt: nuclear mitochondrial
SSCP: single strand conformation polymorphism

## References

[pbio-0020442-Ambrose1] Ambrose SH (1998). Late Pleistocene human population bottlenecks, volcanic winter, and differentiation of modern humans. J Hum Evol.

[pbio-0020442-Avise1] Avise JC, Ball RM (1990). Principles of genealogical concordance in species concepts and biological taxonomy. Oxford Surv Evol Biol.

[pbio-0020442-Bodmer1] Bodmer JG, Marsh SGE, Albert ED, Bodmer WF, Bontrop RE (1999). Nomenclature for factors of the HLA system, 1998. Tissue Antigens.

[pbio-0020442-Boom1] Boom R, Sol CJ, Salimans MM, Jansen CL, Wertheim-van Dillen PM (1990). Rapid and simple method for purification of nucleic acids. J Clin Microbiol.

[pbio-0020442-Clement1] Clement M, Posada D, Crandall KA (2000). TCS: A computer program to estimate gene genealogies. Mol Ecol.

[pbio-0020442-Corbett1] Corbett GB, Hill JE (1993). The mammals of the Indo-Malayan region: A systematic review.

[pbio-0020442-Cracraft1] Cracraft J, Felsenstein J, Vaughn J, Helm-Bychowski K (1998). Sorting out tigers (*Panthera tigris)* Mitochondrial sequences, nuclear inserts, systematics, and conservation genetics. Anim Conserv.

[pbio-0020442-Culver1] Culver M, Johnson WE, Pecon-Slattery J, O'Brien SJ (2000). Genomic ancestry of the American puma *(Puma concolor)*. J Hered.

[pbio-0020442-Dinerstein1] Dinerstein E, Wikramanayake E, Robinson J, Karanth U, Rabinowitz A (1997). A framework for identifying high priority areas and actions for the conservation of tigers in the wild. Part I.

[pbio-0020442-Driscoll1] Driscoll CA, Menotti-Raymond M, Nelson G, Goldstein D, O'Brien SJ (2002). Genomic Microsatellites as evolutionary chronometers: A test in wild cats. Genome Res.

[pbio-0020442-Eizirik1] Eizirik E, Kim JH, Menotti-Raymond M, Crawshaw PG, O'Brien SJ (2001). Phylogeography, population history and conservation genetics of jaguars (*Panthera onca* Mammalia** Felidae). Mol Ecol.

[pbio-0020442-Felsenstein1] Felsenstein J (1989). PHYLIP—Phylogeny inference package (version 3.2). Cladistics.

[pbio-0020442-Fleischer1] Fleischer RC, Perry EA, Muralidharan K, Stevens EE, Wemmer CM (2001). Phylogeography of the Asian elephant *(Elephas maximus)* based on mitochondrial DNA. Evolution.

[pbio-0020442-Foose1] Foose TJ, Tilson RL, Seal US (1987). Species survival plans and overall management strategies. Tigers of the world: The biology, biopolitics, management and conservation of an endangered species.

[pbio-0020442-Fraser1] Fraser DJ, Bernatchez L (2001). Adaptive evolutionary conservation: Towards a unified concept for defining conservation units. Mol Ecol.

[pbio-0020442-Gorog1] Gorog AJ, Sinaga MH, Engstrom MD (2004). Vicariance or dispersal? Historical biogeography of three Sunda shelf murine rodents (Maxomys surifer, Leopoldamys sabanus and *Maxomys whiteheadi)*. Biol J Linn Soc Lond.

[pbio-0020442-Hemmer1] Hemmer H, Tilson RL, Seal US (1987). The phylogeny of the tiger *(Panthera tigris)*. Tigers of the world: The biology, biopolitics, management and conservation of an endangered species.

[pbio-0020442-Hendrickson1] Hendrickson SL, Mayer GC, Wallen EP, Quigley K (2000). Genetic variability and geographic structure of three subspecies of tigers *(Panthera tigris)* based on MHC class I variation. Anim Conserv.

[pbio-0020442-Herrington1] Herrington S, Tilson RL, Seal US (1987). Subspecies and the conservation of Panthera tigris. Tigers of the world: The biology, biopolitics, management and conservation of an endangered species.

[pbio-0020442-Higuchi1] Higuchi R, von Beroldingen CH, Sensabaugh GF, Erlich HA (1988). DNA typing from single hairs. Nature.

[pbio-0020442-Hofreiter1] Hofreiter M, Serre D, Poinar HN, Kuch M, Paabo S (2001). Ancient DNA. Nat Rev Genet.

[pbio-0020442-Hoss1] Hoss M, Paabo S (1993). DNA extraction from Pleistocene bones by silica-based purification method. Nucleic Acids Res.

[pbio-0020442-Hughes1] Hughes JB, Round PD, Woodruff DS (2003). The Indochinese-Sundaic faunal transition at the Isthmus of Kra: An analysis of resident forest bird species distributions. J Biogeogr.

[pbio-0020442-Johnson1] Johnson WE, Dratch PA, Martenson JS, O'Brien SJ (1996). Resolution of recent radiations within three evolutionary lineages of Felidae using mitochondrial restriction fragment length polymorphism variation. J Mammal Evol.

[pbio-0020442-Johnson2] Johnson WE, Slattery JP, Eizirik E, Kim JH, Raymond MM (1999). Disparate phylogeographic patterns of molecular genetic variation in four closely related South American small cat species. Mol Ecol.

[pbio-0020442-Kaplanov1] Kaplanov LG (1948). Tigers in Sikhote-Alin. Tiger, red deer, and moose, Materialy k poznaniyu fauny i flory SSSR.

[pbio-0020442-Kimura1] Kimura M (1955). Solution of a process of random genetic drift with a continuous model. Proc Natl Acad Sci USA.

[pbio-0020442-Kitchener1] Kitchener AC, Seidensticker J, Christie S, Jackson P (1999). Tiger distribution, phenotypic variation and conservation issues. Riding the tiger: Tiger conservation in human-dominated landscapes.

[pbio-0020442-Kitchener2] Kitchener AC, Dugmore AJ (2000). Biogeographical change in the tiger, Panthera tigris. Anim Conserv.

[pbio-0020442-Lister1] Lister AM (2004). The impact of Quaternary Ice Ages on mammalian evolution. Philos Trans R Soc Lond B Biol Sci.

[pbio-0020442-Lopez1] Lopez JV, Cevario S, O'Brien SJ (1996). Complete nucleotide sequences of the domestic cat *(Felis catus)* mitochondrial genome and a transposed mtDNA tandem repeat *(Numt)* in the nuclear genome. Genomics.

[pbio-0020442-Lopez2] Lopez JV, Yuhki N, Masuda R, Modi W, O'Brien SJ (1994). Numt, a recent transfer and tandem amplification of mitochondrial DNA to the nuclear genome of the domestic cat. J Mol Evol.

[pbio-0020442-Maguire1] Maguire LA, Lacy RC (1990). Allocating space resources for conservation of endangered subspecies: Partitioning zoo space for tigers. Conserv Biol.

[pbio-0020442-Matyushkin1] Matyushkin EN, Pikunov DG, Dunishenko YM, Miquelle DG, Nikolaev IG, Aristova AA (1999). Distribution and numbers of Amur tigers in the Russian Far East in the mid-1990's. Rare mammal species of Russia and neighboring territories (in Russian).

[pbio-0020442-Mazak1] Mazak V (1981). Panthera tigris. Mamm Species.

[pbio-0020442-Mazak2] Mazak V (1996). Der Tiger.

[pbio-0020442-Mazak3] Mazak V (1968). Tigre provenant de r Asiedu Sud-Est. Mammalia.

[pbio-0020442-Menotti-Raymond1] Menotti-Raymond M, David VA, Lyons LA, Schaffer AA, Tomlin JF (1999). A genetic linkage map of microsatellites in the domestic cat *(Felis catus)*. Genomics.

[pbio-0020442-Menotti-Raymond2] Menotti-Raymond M, David VA, Chen ZQ, Menotti KA, Sun S (2003). Second-generation integrated genetic linkage/radiation hybrid maps of the domestic cat *(Felis catus)*. J Hered.

[pbio-0020442-Minch1] Minch E, Ruiz-Linares A, Goldstein DB (1995). MICROSAT. Available: http://hpgl.stanford.edu/projects/microsat/ via the Internet. http://hpgl.stanford.edu/projects/microsat/.

[pbio-0020442-Miquelle1] Miquelle DG, Pikunov DG, Newell JP (2003). Status of the Amur tiger and Far Eastern leopard. The Russian Far East: A reference guide for conservation and development.

[pbio-0020442-Moritz1] Moritz C (1994). Defining evolutionarily significant units for conservation. Trends Ecol Evol.

[pbio-0020442-Nei1] Nei M (1987). Molecular Evolutionary Genetics.

[pbio-0020442-Nowell1] Nowell K, Jackson P (1996). Wild cats: Status survey and conservation action plan.

[pbio-0020442-OaBrien1] O'Brien SJ, Collier GE, Benveniste RE, Nash NG, Newman AK, Tilson RL, Seal US (1987). Setting the molecular clock in Felidae: the great cats *Panthera*. Tigers of the world: The biology, biopolitics, management and conservation of an endangered species.

[pbio-0020442-OaBrien2] O'Brien SJ, Mayr E (1991). Bureaucratic mischief: Recognizing endangered species and subspecies. Science.

[pbio-0020442-Posada1] Posada D, Crandall KA (1998). MODELTEST: Testing the model of DNA substitution. Bioinformatics.

[pbio-0020442-Pritchard1] Pritchard JK, Stephens M, Donnelly P (2000). Inference of population structure using multilocus genotype data. Genetics.

[pbio-0020442-Rampino1] Rampino MR, Self S (1992). Volcanic winter and accelerated glaciation following the Toba super-eruption. Nature.

[pbio-0020442-Raymond1] Raymond M, Rosset F (1995). GENEPOP (Version 1.2): Population genetics software for exact test and ecumenicism. J Hered.

[pbio-0020442-Rogers1] Rogers AR, Harpending H (1992). Population growth makes waves in the distribution of pairwise genetic differences. Mol Biol Evol.

[pbio-0020442-Rozas1] Rozas J, Sanchez-DelBarrio JC, Messeguer X, Rozas R (2003). DnaSP, DNA polymorphism analyses by the coalescent and other methods. Bioinformatics.

[pbio-0020442-Sambrook1] Sambrook J, Fritsch E, Maniatis T (1989). Molecular Cloning: a Laboratory Manual.

[pbio-0020442-Schneider1] Schneider S, Roessli D, Excoffier L (2000). Arlequin: A Software for Population Genetics Data Analysis.

[pbio-0020442-Seidensticker1] Seidensticker J, Christie S, Jackson P (1999). Riding the Tiger: Tiger Conservation in Human-Dominated Landscapes.

[pbio-0020442-Smith1] Smith JLD, McDougal C (1991). The Contribution of Variance in Lifetime Reproduction to Effective Population Size in Tigers. Conserv Biol.

[pbio-0020442-Swofford1] Swofford DL (2001). ‘PAUP* Phylogenetic Analysis Using Parsimony and Other Methods' Computer Program.

[pbio-0020442-Takezaki1] Takezaki N, Rzhetsky A, Nei M (1995). Phylogenetic test of the molecular clock and linearized trees. Mol Biol Evol.

[pbio-0020442-Thompson1] Thompson JD, Gibson TJ, Plewniak F, Jeanmougin F, Higgins DG (1997). The CLUSTAL-X windows interface: flexible strategies for multiple sequence alignment aided by quality analysis tools. Nucleic Acids Res.

[pbio-0020442-Tilson1] Tilson R, Nyhus P, Franklin F, Maher D, Noss R, Larkin J (2001). Tiger Restoration in Asia: Ecological Reality and Sociological Reality. Large Mammal Restoration: Ecological and Sociological Challenges in the 21st Century: Island Press.

[pbio-0020442-Tilson2] Tilson R, Defu H, Muntifering J, Nyhus PJ (2004). Dramatic decline of wild South China tigers *Panthera tigris amoyensis*: field survey of priority tiger reserves. Oryx.

[pbio-0020442-Tosi1] Tosi AJ, Morales JC, Melnick DJ (2002). Y-chromosome and mitochondrial markers in Macaca fascicularis indicate introgression with Indochinese M-mulatta and a biogeographic barrier in the Isthmus of Kra. Int J Primatol.

[pbio-0020442-Turner1] Turner A, Anton M (1997). The Big Cats and their Fossil Relatives. An illustrated guide to their evolution and natural history.

[pbio-0020442-Uphyrkina1] Uphyrkina O, Johnson WE, Quigley H, Miquelle D, Marker L (2001). Phylogenetics, genome diversity and origin of modern leopard, Panthera pardus. Mol Ecol.

[pbio-0020442-Wayne1] Wayne RK, Van Valkenburgh B, O'Brien SJ (1991). Molecular Distance and Divergence Time in Carnivores and Primates. Mol Biol Evol.

[pbio-0020442-Wentzel1] Wentzel J, Stephens C, Johnson WE, Menotti-Raymond M, Pecon-Slattery J, Seidensticker J, Christie S, Jackson P (1999). Subspecies of tigers: molecular assessment using “voucher specimens” of geographically traceable individuals. Riding the Tiger: Tiger Conservation in Human-Dominated Landscapes.

[pbio-0020442-Woodruff1] Woodruff DS (2003). Neogene marine transgressions, palaeogeography and biogeographic transitions on the Thai-Malay Peninsula. J Biogeogr.

[pbio-0020442-Yuhki1] Yuhki N, O'Brien SJ (1997). Nature and origin of polymorphism in feline MHC class II DRA and DRB genes. J Immunol.

[pbio-0020442-Yuhki2] Yuhki N, Beck T, Stephens RM, Nishigaki Y, Newmann K (2003). Comparative genome organization of human, murine, and feline MHC class II region. Genome Res.

