# Supplementary material for: Phylogeography and Genetic Ancestry of Tigers (Panthera tigris)
Source: PLoS Biol. 2004 Dec 7;2(12):e442. doi: 10.1371/journal.pbio.0020442 (PMC534810; doi:10.1371/journal.pbio.0020442)
Supplement: Figure S1 — Branches of the same color represent tiger individuals of the same classically named subspecies. NJ tree constructed based on kinship coefficient (Dkf) with the (1 – kf) option in MICROSAT (Minch et al. 1995). Numbers are individual Pti codes (Table 3). Bootstrap values over 50% are shown on divergence nodes. (108 KB DOC). [file pbio.0020442.sg001.doc]

0.1

158

161

128

127

111

134

151

159

143

123

118

112

131

157

122

154

142

148

145

133

124

114

115

137

138

152

156

120

117

126

135

147

113

146

103

102

104

105

316

222

217

218

290

291

CB36

CB34

CB31

CB32

CB14

CB6

CB23

CB11

CB18

CB13

CB24

CB16

CB7

219

220

99

184

150

216

206

209

171

172

174

181

185

178

183

186

170

175

90

165

297

296

301

315

249

305

306

307

CB15

CB22

263

304

108

269

163

270

211

273

210

272

265

266

268

267

247

271

255

303

253

254

262

264

250

CB29

292

CB27

76

99

*P. t. corbetti* II

(*P. t. jacksoni*)

*P. t. altaica*

*P. t. corbetti* I

*P. t. amoyensis*

*P. t. sumatrae*

*P. t. tigris*

Dkf

Supplementary Material 1. Phylogenetic relationships among the individual tigers from composite microsatellite genotypes of 30 loci. Branches of the same color represent tiger individuals of the same classically named subspecies. Neighbor-joining tree constructed based on kinship coefficient (Dkf) with (1-kf) option in MICROSAT (Minch et al. 1995). Numbers are individual Pti codes (Table 1). Bootstrap values over 50% are shown on divergence nodes.
