# Supplementary material for: Phylogeography and Genetic Ancestry of Tigers (Panthera tigris)
Source: PLoS Biol. 2004 Dec 7;2(12):e442. doi: 10.1371/journal.pbio.0020442 (PMC534810; doi:10.1371/journal.pbio.0020442)
Supplement: Figure S2 — Data obtained from microsatellite genotype and mitochondrial haplotype data were analyzed using STRUCTURE (Pritchard et al. 2000). Simulations were set at 50,000 burn-in period followed by 106 replicates. Each individual is represented by a thin vertical bar, which is partitioned into K colored segments that represent the individual affiliation to each of K clusters. Here shows the population structure when K = 7, which produced the highest probability among other choices of K. Three STRUCTURE runs produced almost identical individual affiliation. (62 KB DOC). [file pbio.0020442.sg002.doc]

ALT

SUM

TIG

CORII

CORI

Figure S2. Bayesian Population Structure Analysis of 111 Tigers

Data obtained from microsatellite genotype and mitochondrial haplotype data were analyzed using STRUCTURE (Pritchard et al. 2000). Simulations were set at 50,000 burn-in period followed by 10^6^ replicates. Each individual is represented by a thin vertical bar, which is partitioned into *K* colored segments that represent the individual affiliation to each of *K* clusters. Here shows the population structure when *K* = 7, which produced the highest probability among other choices of *K*. Three STRUCTURE runs produced almost identical individual affiliation.
