# Supplementary material for: Phylogeography and Genetic Ancestry of Tigers (Panthera tigris)
Source: PLoS Biol. 2004 Dec 7;2(12):e442. doi: 10.1371/journal.pbio.0020442 (PMC534810; doi:10.1371/journal.pbio.0020442)
Supplement: Table S1 — (59 KB DOC). [file pbio.0020442.st001.doc]

Table S1.

| Bayesian Clustering Analyses for Tiger Microsatellite and Mitochondrial Data^*^ | | | | |
| --- | --- | --- | --- | --- |
| K | Ln probability of K clusters | Probability of K clusters | Variance Ln | Alpha |
| 1 | -9733 | ~0 | 95.1 | - |
| 2 | -8473 | ~0 | 177.1 | 0.00352 |
| 3 | -7811 | ~0 | 276.1 | 0.00318 |
| 4 | -7197 | ~0 | 369.1 | 0.00309 |
| 5 | -6870 | ~0 | 391.6 | 0.00281 |
| 6 | -6733 | ~0 | 481.1 | 0.00277 |
| 7 | **-6595** | **0.993** | 550.0 | 0.00274 |
| 8 | -6624 | ~0 | 690.8 | 0.00273 |
| 9 | -6600 | 0.007 | 750.2 | 0.00273 |
| 10 | -6677 | ~0 | 1006.7 | 0.00270 |
| 11 | -6862 | ~0 | 1422.4 | 0.00269 |

*. A total of 111 individuals analyzed at 30 microsatellite loci and mtDNA sequence (considered as one haploid locus) were assigned to clusters using STRUCTURE (Pritchard et al. 2000). The number of clusters is indicated by K. Probability of the data are in the Ln probability and probability of K clusters columns and the variance of the probability is presented in the variance Ln column. Alpha values indicate the admixture value. Lowest K is in bold type.
